# Predator crow search optimization with explainable AI for cardiac vascular disease classification

**DOI:** 10.1038/s41598-025-96003-9

**Published:** 2025-04-05

**Authors:** M. M. Asha, G. Ramya

**Affiliations:** School of Computer Science Engineering and Information Systems, Vellore Institute of Technology, Vellore, 632014 Tamilnadu India

**Keywords:** Cardiovascular diseases (CVDs), Left ventricle (LV), Predator crow search optimization (PCSO), Explainable AI (XAI) algorithm, Modified U-Net, Cardiology, Diseases, Medical research

## Abstract

The proposed framework optimizes Explainable AI parameters, combining Predator crow search optimization to refine the predictive model’s performance. To prevent overfitting and enhance feature selection, an information acquisition-based technique is introduced, improving the model’s robustness and reliability. An enhanced U-Net model employing context-based partitioning is proposed for precise and automatic left ventricular segmentation, facilitating quantitative assessment. The methodology was validated using two datasets: the publicly available ACDC challenge dataset and the imATFIB dataset from internal clinical research, demonstrating significant improvements. The comparative analysis confirms the superiority of the proposed framework over existing cardiovascular disease prediction methods, achieving remarkable results of 99.72% accuracy, 96.47% precision, 98.6% recall, and 94.6% F1 measure. Additionally, qualitative analysis was performed to evaluate the interpretability and clinical relevance of the model’s predictions, ensuring that the outputs align with expert medical insights. This comprehensive approach not only advances the accuracy of CVD predictions but also provides a robust tool for medical professionals, potentially improving patient outcomes through early and precise diagnosis.

## Introduction

Globally, Cardiovascular Disease is one of the primary causes of significant illness and mortality. Myocardial infarction, stroke, and vertigo are symptoms of cardiovascular illness, which is a malfunction of the circulatory system^1^. It has been demonstrated that several risk factors, including high cholesterol, high blood pressure, and diabetes, affect cardiovascular disease. Most of the time, early cardiac disease is still difficult to diagnose despite ongoing technological advancements^2^. Table 1 provides the list of acronyms and their abbreviations.

**Table 1:** List of acronyms and abbreviations.

Acronyms	Abbreviation
ACDC	Automated Cardiac Diagnosis Challenge
CVD	Cardiovascular Disease
DCM	Dilated Cardiomyopathy
HCM	Hypertrophic Cardiomyopathy
IGFS	Information Gain-based Feature Selection
LV	Left Ventricle
MINF	Myocardial Infarction
MRI	Magnetic Resonance Imaging
PCSO	Predator Crow Search Optimization
SMOTE	Synthetic Minority Over-sampling Technique
XAI	Explainable Artificial Intelligence

Heart disease can significantly impair a person’s health and productivity^3^. A quick slump to the ground may result in sudden cardiac arrest. During cardiac arrest, medical devices like defibrillators shock the heart with tremendous energy, aiding in the patient’s recovery and restoration of normal cardiac function. According to the World Health Organization (WHO), throughout the previous several decades, chronic illnesses have dramatically grown in developed countries^4^. The aging population and illnesses linked to lifestyle choices are the major causes of this. Another significant aspect that makes treating these people more difficult is co-morbidity, or having many diseases in one person. Elderly people’s comorbid illnesses should receive greater focus. The therapy and prevention of these illnesses have made greater use of innovative technology in recent years^5,6^.

A diagnosis relies on a wide range of health-related data, which is reflected in the large number of attributes within the dataset^7^. Each feature plays a unique role in identifying the presence of a disease. Feature selection techniques can be applied before training the model to extract the most relevant characteristics, enabling faster and more accurate predictions. However, most disease datasets are imbalanced, meaning that the number of positive cases is significantly lower than negative ones. This imbalance can impact model performance, but data distribution can be adjusted using specific preprocessing methods to enhance effectiveness^8^.

**Fig. 1:**
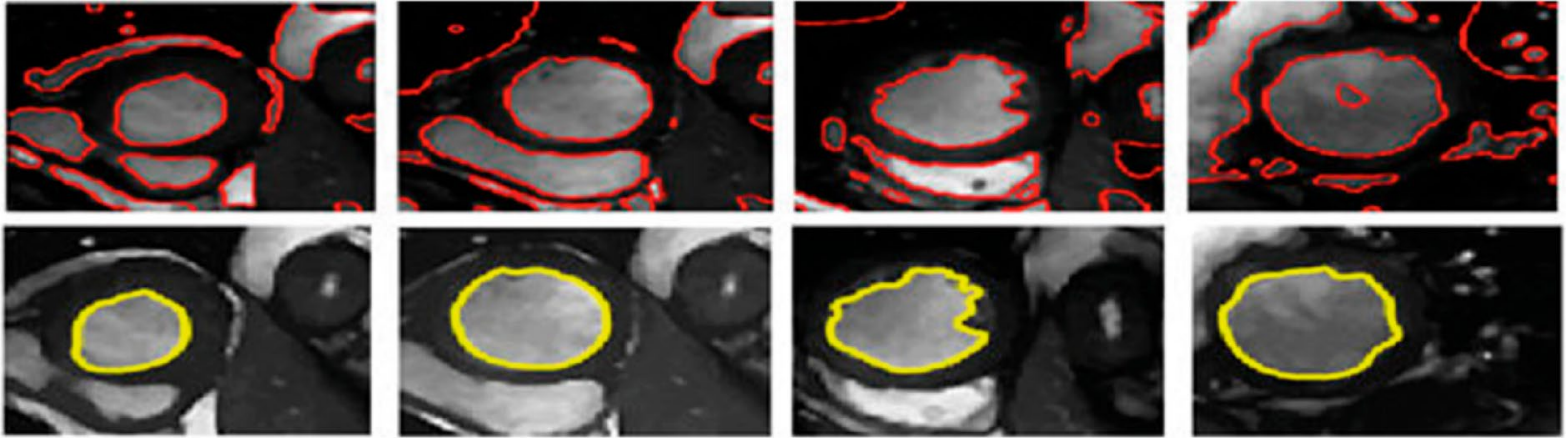
Images in the second-row exhibit corresponding manual segmentation, while images in the first row display erroneous automated segmentation.

The automatic segmentation of the left ventricular myocardium encompasses several key research areas. Significant progress and contributions have been made in this field. Various models support segmentation, including deformable models based on pixel representations, statistical approaches, and heart map-based techniques^9^. Studies have shown that deep learning methods outperform traditional techniques, yielding remarkable anatomical results^10,11^. However, errors can still occur, making manual separation challenging. Figure 1 illustrates an example of incorrect segmentation and its impact. While a convolutional neural network was used to generate the initial segmented image, the experts referenced in^12^ manually refined the second row of images.

Patients can keep an eye on their health with wearable or portable equipment if necessary. They can consult virtual assistants for medical advice and use remote facilities to operate their dwellings. Highly developed clinical decision support systems, according to medical professionals, can be utilized to direct and enhance medical tests. The notion of the “Internet of Medical Things” (IoMT) has gained traction as healthcare professionals increasingly adopt and utilize contemporary clinical equipment and related hardware^13^. Researchers and medical practitioners have new opportunities as a result of the significant changes taking place in the medical business and the adoption of IoT-assisted medical devices^14^. Thanks to this development, researchers may now track smartphone usage and device behaviors by utilizing a range of sensors, including built-in sensors, ingestible devices, and portable ones to monitor user activity. The abundance of data gathered enables us to make use of contemporary technologies like deep learning and artificial intelligence to understand more about a person’s health^15^. Deep learning approaches in machine learning have demonstrated great potential in population-based research for the identification of useful biomarkers like ECG signals, the prediction of cardiac events, and the assessment of cardiovascular risk^16,17^.

Though they have substantial drawbacks, a number of machine learning-based techniques for detecting and forecasting cardiac disease have recently surfaced. It is frequently difficult for traditional intelligence frameworks to use data from numerous sources efficiently, particularly when working with high-dimensional data sets^18^. Furthermore, the accuracy of diagnosing cardiac disease is not always increased by the existing algorithms, which usually choose variables from a dataset and determine their overall relevance. Additionally, existing optimization techniques often struggle with feature selection, leading to suboptimal model performance.

### Problem statement

The effectiveness of CVD prediction models is often affected by several challenges:

- Most ML models operate as black-box systems, making it difficult for healthcare professionals to trust their predictions.
- Traditional optimization techniques do not always identify the most relevant features, reducing model accuracy.
- Existing deep learning-based segmentation methods lack robustness in detecting left ventricular abnormalities.
- Many CVD datasets suffer from class imbalances, which can bias model predictions and reduce reliability.

### Motivation

To address these limitations, this study introduces a hybrid approach integrating Predator Crow Search Optimization with Explainable AI techniques to improve CVD classification. Unlike conventional deep learning models, our framework optimizes feature selection and classifier parameters using PCSO, ensuring enhanced model accuracy and efficiency. Furthermore, we propose an enhanced U-Net model for left ventricular segmentation, improving the precision of cardiac MRI analysis. This hybrid approach not only increases the reliability of predictions but also offers interpretability, making it suitable for clinical applications.The following is an overview of the study’s main findings.

- Integration of PCSO with XAI for CVD classification, enhancing both accuracy and interpretability.
- Optimized Feature Selection using PCSO to identify the most relevant predictors, reducing computational complexity.
- Improved Cardiac MRI Segmentation using a modified U-Net architecture, leading to more precise left ventricular analysis.
- Handling Data Imbalance through the application of oversampling techniques such as SMOTE, ensuring a balanced dataset.
- Comparative Performance Analysis with existing models, demonstrating superior accuracy, precision, recall, and F1-score.

The remainder of this work is structured as follows: Sect. "Literature survey" reviews existing methodologies for CVD prediction and segmentation. Section "Materials and methods" describes the methodology. Section "Performance metrics" presents the performance metrics. Section "Results and discussion" details results and discussion. Section "Experimental findings" discusses experimental results and performance comparisons. Section "Limitations and future directions" presents limitations with future research directions. Finally Sect. "Conclusion" concludes the study. This research contributes to the field of medical AI by addressing the interpretability and efficiency challenges in CVD prediction, paving the way for more reliable and explainable clinical decision support systems.

## Literature survey

A variety of models and methodologies, each with its own set of drawbacks, have been created over time in the field of cardiovascular disease prediction. These constraints dictate the necessity for the proposed model, which attempts to resolve these problems. The data and experiences of medical experts are the sources of knowledge in the medical field^19^. It is difficult and time-consuming to simulate the functioning and dysfunctions of the human body due to its complexity and susceptibility to several influences. In electronic medical systems, machine learning technology is crucial for the diagnosis and prognosis of various diseases based on medical data^20^. For instance, Mansour et al. presented the Crow Search Optimization-based Cascaded Long Short-Term Memory (CSO-CLSTM) framework in prior research^21^ to use fusion and artificial intelligence techniques to diagnose disorders. Strong specificity and classification rate are demonstrated by the CSO-CLSTM model. Nevertheless, we ran into issues because of the suggested system’s intricacy^22^.

As demonstrated in the work of^23^, an improved three-tier system provides another method for handling substantial volumes of data from wearable sensors. Layer 1 concentrates on data gathered from wearable IoT sensors; Layer 2 stores data from cloud-integrated IoT devices using Apache HBase; and Layer 3 stores data from probabilistic linear. An external prediction model for advancing heart disease is developed. However, because this method is sequential, it may be computationally challenging. Munagala et al.^24^ presented an innovative strategy to increase the prediction accuracy of cardiovascular illness by identifying key traits using machine learning techniques. To create predictive models with better performance, several elements and established techniques are combined. This cutting-edge technology is easy to use, effective, and less expensive, and it enhances heart disease prognosis. Feature selection methods are required to obtain a more thorough understanding of relevant data in order to improve the prediction accuracy of cardiac disease.

Balamurugan et al.^25^ created deep learning techniques with ECG recordings lasting five minutes. They used ECG data to search for time-frequency features that could be used to predict vascular incidents many days in advance. We examined the concept of recognising long-term relationships and having the ability to quickly recognise and put an end to these instances using LSTM (long short-term memory) neural networks. Nonetheless, this study’s weaknesses include the topic’s distinctiveness and the requirement for precise experimental and assessment criteria.

Using a continuous remote patient monitoring program^26^, created a hybrid fuzzy tree-based method for cardiac illness early detection.The hybrid fuzzy-based decision tree approach has demonstrated superiority over prior classification techniques in the accurate detection of heart disease. For the deployment of IoT, there isn’t a clear system or set of standards, though. This isn’t available everywhere, but it ought to offer a suitable resolution to the issue. An IoT-based medical system using a random forest classifier was created by^24^. The system that has been devised enhances communication between physicians and patients. Nevertheless, creating and implementing mobile-based healthcare systems comes with several difficulties. Researchers in^27^ recently introduced a system for tracking health using deep learning and the Internet of Things. Among athletes, this technique may be useful in detecting potentially harmful conditions including tumors, heart problems, cancer, etc.

Nevertheless, the classifier used to build the model could lead to overfitting, complexity, and costly processing. For instance^28^, created an Internet of Things-based smart sports wristband system that tracks heart rate. Monitor heart rate variations when patients exercise. Heart rate is the primary focus of the model’s physiological parameters. Blood pressure is one important sign that has to be monitored. This is the main problem with this strategy.

Recent advancements in optimization techniques have significantly improved energy efficiency in various domains, including fuel cell electric vehicles (FCEVs). A notable study^29^ integrates the Siberian Tiger Optimization (STO) algorithm with the Improved Wasserstein Generative Adversarial Network (IWGAN) to enhance energy management in hybrid battery systems. The STO method optimizes energy allocation, while the IWGAN model accurately predicts power demand.

The researcher in^30^ proposes a machine learning-based method for early heart disease diagnosis using the Cleveland heart disease dataset. The study employs the Random Forest algorithm for classification, leveraging 14 physiological attributes and validating performance through 10-Fold Cross-Validation. The model achieves an accuracy of 90.16%, outperforming several existing techniques. The results highlight the effectiveness of RF in heart disease prediction, demonstrating its potential for clinical decision support.

The work in^31^ presents a hybrid XGBoost Classifier framework for heart disease prediction, incorporating outlier removal using z-score and hyperparameter optimization with Optuna. It evaluates the impact of different train-test splits (70:30, 80:20, 90:10) on model performance using the Cleveland HD dataset. The proposed model achieves 95.45% accuracy with optimized preprocessing and hyperparameters, validated through Stratified K-Fold Cross-Validation, offering an efficient solution for accurate HD diagnosis.The following Table 2 summarizes key research studies, their datasets, methodologies, optimization techniques, accuracy, and research gaps.

**Table 2:** Existing literature: contributions and research gaps.

Ref	Dataset	Methodology	Optimization Technique	Accuracy	Research Gap
^32^	UCI Heart Disease	HyOPTXGBoost and HyOPTRF	Optuna Optimization	94.2%	Lack of interpretability
^33^	Cleveland	Supervised ML Algorithms	Traditional ML	92.8%	Limited feature selection optimization
^34^	Framingham	Hybrid XGBoost with Optuna tuning	Hyperparameter tuning	96.1%	High sensitivity to outliers
^35^	MIT-BIH Arrhythmia	ML algorithms	Feature Selection	93.7%	Computationally expensive
^36^	UCI Heart Disease	Genetic Algorithm	GA	95.3%	High training time
^37^	ECG-based Dataset	CNN-LSTM	CNN-LSTM	92.4%	High computational cost
^38^	Custom CVD Dataset	Deep Reinforcement Learning for heart disease detection	DRL	90.9%	Slow convergence
^39^	UCI Heart Disease	Gradient-Based Optimizer	GBO	95.1%	Sensitive to parameter selection
^40^	ECG-based Dataset	Rime Optimization Algorithm for ECG-based detection	RIME	91.7%	High computational cost
^41^	MIT-BIH Dataset	Heap-Based Optimizer (HBO) for ML-based heart disease detection	HBO	93.2%	Lack of interpretability

Despite significant advancements in CVD prediction and cardiac MRI segmentation, existing methods face several challenges, including lack of interpretability, suboptimal feature selection, inefficient segmentation techniques, high computational costs, and class imbalance issues. Many deep learning models act as black boxes, limiting their clinical trustworthiness, while traditional optimization techniques such as Genetic Algorithms and Particle Swarm Optimization often suffer from slow convergence. Additionally, current feature selection methods may not always capture the most relevant predictors, leading to reduced model performance. To address these limitations, our work introduces a hybrid PCSO-XAI framework that integrates Predator Crow Search Optimization and Explainable AI for model transparency, and a modified U-Net for improved left ventricular segmentation.

## Materials and methods

### Study approval and participants

The study obtained ethical approval from the Institutional Ethics Committee, Vellore Institute of Technology (Nr.20117/04.10.2016) and recruited subjects between 2017 and 2020. All methods were performed in accordance with the relevant guidelines and regulations of the local ethics committee. Written informed consent permission was provided by each participant to take part in the research.

**Fig. 2:**
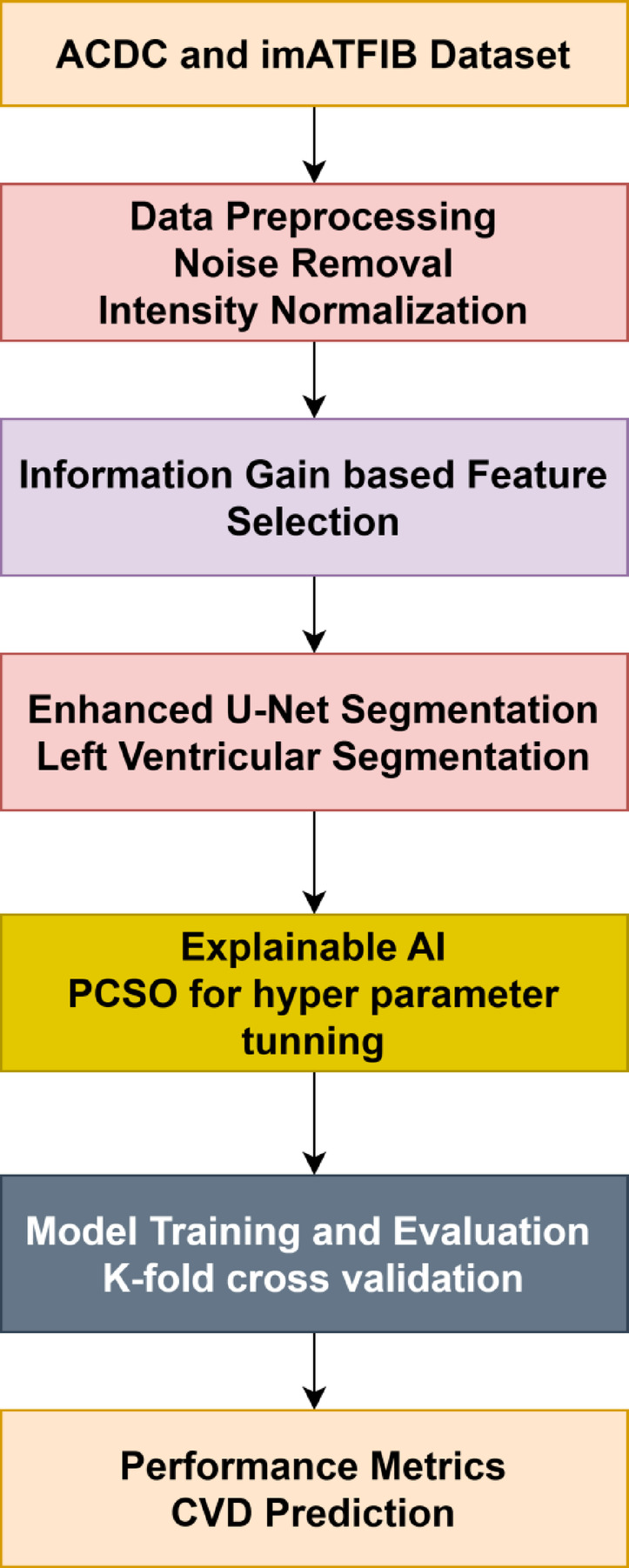
Overall Proposed Flow.

The architecture, data preprocessing, feature selection, segmentation, and classification are the essential elements of the proposed method that are covered in this section. The PCSO-XAI and U-Net architecture is employed for tasks involving segmentation and classification. Once the network has undergone preprocessing stage, input images are fed into it, and it is trained to anticipate the semantic labels connected to each image voxel. The model is only subjected to the chosen area of the image when it is chosen using the information capture function. Figure 2 depicts the overall proposed flow of the study.

### Datasets

#### ACDC dataset

There are 150 diastolic and systolic truncated cine MRI volumes accessible in the ACDC challenge^4^, of which 100 are set aside for testing and training and the remaining 50 for validation. This dataset provides the segmentation masks for the left ventricle (LV), myocardium (Myo), and right ventricle (RV). ACDC dataset is publicly available at https://www.creatis.insa-lyon.fr/Challenge/acdc/.

#### imATFIB Dataset

The data set originates from a self-conducted clinical research called imATFIB (Continuous Non-Invasive Atrial Fibrillation Diagnosis using IMaging). ClinicalTrials.gov (accessed February 22, 2021) has the study registered under registration number NCT03584126. The regional ethics council granted ethical permission for this study (No. 20117/04.10.2016), and participants were gathered for the research between 2017 and 2020. Written informed consent permission was provided by each participant to take part in the research. After electrocardiography and echocardiography were used to examine the heart in patients and healthy subjects, cardiac MRI measures were performed.

Using specialized body coils for signal reception, a 3.0T Discovery MR750w General Electric MRI scanner was deployed to image all individuals. To get around motion artifacts in cardiac MRI, cardiac gating and breath-holding methods synced with the ECG are necessary. A post-contrast sequence, a FIESTA CINE sequence (ALL FIESTA CINE AST), and a dark blood sequence (Black Blood SSFSE) make up the cardiac MRI procedure. Construct pictures using the perspective, 4-chamber, and 2-chamber perspectives from three separate planes (2D MDE, 2D PSMDE). A 10-minute interval following gadolinium administration is used to acquire an MDE/LGE sequence that contrasts the normal (dark colored) and aberrant (enhanced) myocardium. Additionally, coronary artery abnormalities were assessed with the acquisition of 3D HEART sequences. Throughout the testing process, participants were closely observed, and no adverse effects were recorded right away. Table 3 provides the details of the ACDC and imATFIB datasets.

**Table 3:** Dataset characteristics and balancing techniques used in the study.

Dataset	Samples	Classes	Class Type	Balancing Technique
ACDC	150 cine MRI volumes	4 (Normal, MINF, DCM, HCM)	Multi-class	No balancing needed (already balanced)
imATFIB	20 cardiac MRI volumes	2 (Healthy, Atrial Fibrillation)	Binary	**SMOTE** used for balancing

Two radiologists manually tracked the heart in ten datasets, ten from patients with atrial fibrillation and ten from healthy participants. A senior radiologist with over twenty years of expertise verified the findings after they had been manually annotated by a radiologist with over ten years of experience.The DICOM format is used to export and store the results. Using whole-heart segmentation masks, 20 cardiac MRI volumes were obtained as part of the independently carried out clinical research imATFIB. The ACDC dataset is recognized as a reliable benchmark since its resolution (256 × 256 × 256 for imATFIB), slice spacing, and slice thickness are similar to those of the imATFIB dataset. This dataset requires permission (NCT03584126).

### Data pre-processing

Data preprocessing is a crucial stage in machine learning that seeks to enhance the calibre and dependability of data gathering prior to analysis and modelling. Missing data, inconsistent data, outliers, and skewed class distributions are all addressed in this stage. In order to use strategies like replacement to guarantee correct insights, it is imperative to resolve missing data. Outliers can distort outcomes, thus it’s critical to identify and handle them. The primary goal is to achieve balance in the class distribution, which can help to mitigate dataset imbalances such as oversampling. In order to optimize data for efficient machine learning analysis, methods including dimensionality reduction, augmentation, and standardization can be applied.

To ensure high-quality input data for the proposed model, a comprehensive data cleaning and normalization process was implemented. Noise removal was performed using Gaussian filtering, which effectively reduces random noise and artifacts present in MRI scans, enhancing image clarity without compromising structural integrity. Additionally, intensity normalization was applied using Z-score normalization, where pixel intensity values were standardized to have a mean of zero and a unit standard deviation. This normalization step ensured uniform contrast and brightness across different MRI scans, minimizing the variability caused by differences in imaging equipment and acquisition conditions. Furthermore, outlier detection techniques were employed to identify and remove abnormal signal variations that could negatively impact model performance. This was achieved using statistical methods, where pixel intensities deviating significantly from the normal distribution were flagged and corrected. These data cleaning and normalization steps played a crucial role in improving the robustness and reliability of the dataset.

To prevent overfitting and bias, several techniques were implemented throughout the study. Feature selection using the Information Gain-based Feature Selection method was applied to eliminate redundant and less informative attributes, reducing model complexity and improving generalization. The U-Net model utilized batch normalization and a 10% dropout rate in its convolution layers to prevent overfitting by regularizing the network and ensuring it does not memorize patterns from the training data. Additionally, hyperparameter tuning using the Predator Crow Search Optimization algorithm allowed for optimal parameter selection, enhancing the model’s robustness and preventing overfitting. An 80:20 train-test split was employed for model evaluation, ensuring reliable validation. To handle dataset imbalance, oversampling techniques were applied to increase the representation of the minority class, particularly for cardiovascular disease cases. Data augmentation, including rotation, flipping, and contrast enhancement, was used to enhance MRI image diversity, further improving the model’s ability to generalize. Moreover, a weighted loss function was incorporated to assign higher penalties to the minority class, preventing bias toward the majority class. Additionally, qualitative analysis was incorporated by leveraging expert evaluations of segmentation outputs and model predictions to assess their alignment with real-world clinical interpretations. These combined techniques ensure that the proposed framework generalizes well, avoids overfitting, and maintains a balanced classification approach for cardiovascular disease prediction.

### Feature selection based on information gain

When choosing features that significantly affect the outcome, HDD uses an information gain-based feature selection technique^15^ to weed out redundant and pointless characteristics. Algorithm 1 uses the pseudocode of IGFS.

**Algorithm 1:**
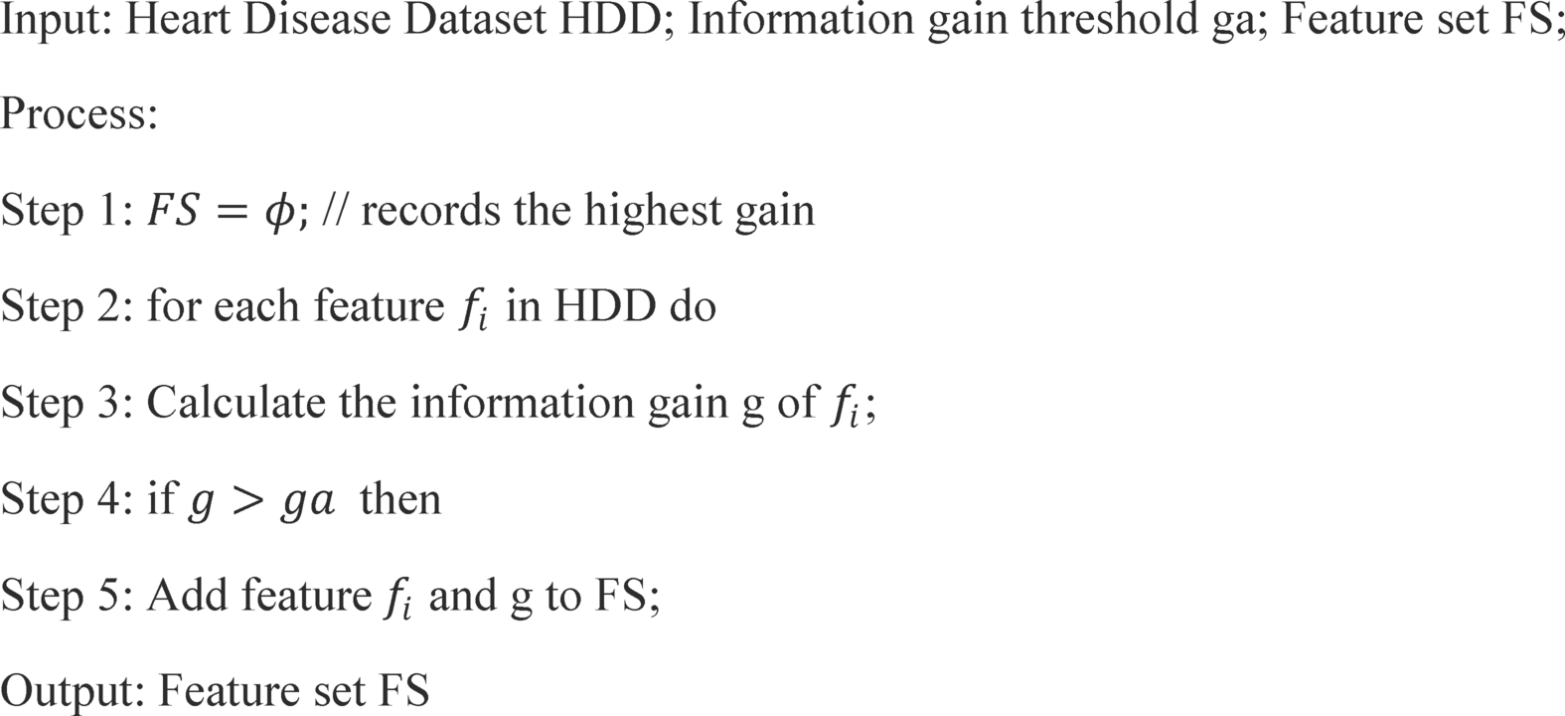
The pseudocode of IGFS.

Information from certain trait sets is utilized as input characteristics for prediction in order to decrease trait dimensionality and enhance model predictions. The primary task-related properties must be preserved during feature selection, and this study uses an information-acquisition-based feature selection approach. This method’s primary goal is to measure a feature’s significance from the standpoint of information acquisition. The information retrieval rate increases with the amount of feature information that aids in categorization. The formula to calculate gain is as follows.

1 $$\:E\left(X\right)=-\sum\:_{i}{p}_{i}{\text{log}}_{2}{p}_{i}$$

Where,

E(X) represents the entropy of the dataset X.

Σ represents the probability of an element belonging to class ‘i’.

$\:{\:\:\:\:\:\:\:\:\:\:\:\:\:\:p}_{i}\:$denotes the sum of the following calculations for each class.

$\:\:\:\:\:\:\:\:\:\:\:\:\:\:\:{\text{log}}_{2}{p}_{i}$is the base-2 logarithm of p(i).

2 $$\:E\left(\frac{Y}{X}\right)=P\left(X=v\right)E\left(\frac{Y}{X+v}\right)$$

Where,

Y/X: Random Variable.

P(X = v): Probability of X being v.

v: Specific Value

3 $$\:IG\left(X,Y\right)=E\left(Y\right)-E\left(\frac{Y}{X}\right)$$

Where,

IG(X, Y): Information Gain.

E(Y): Entropy of Y.

E(Y/X): Conditional Entropy of Y given X.

The difference between information entropy and conditional entropy is the feature. It ranks these values by using various information gain values for various dataset attributes. Characteristics are deemed fundamental characteristics that must be chosen if their benefit exceeds the threshold. Following the aforementioned feature selection and preprocessing, the resulting dataset comprised 3527 samples, each with 15 characteristics and 1 projected label. The preprocessed HDD is detailed in Table 4 below.

**Table 4:** Description of feature.

S.No	Feature	Type	Description
1	Sex	Categorical	Male = 1, Female = 0
2	Stable_CAD	Categorical	Stable CAD = 0, Unstable CAD = 1
3	Age	Numeric	Age in years [20:86]
4	CVD_history	Categorical	Ischemic cerebro vascular disease = 0, Hemorrhagic cerebral vascular disease = 1
5	Smoke	Categorical	No smoking = 0, Smoking = 1
6	Nitrate	Categorical	Without nitrate = 0, with nitrate = 1
7	LVEF	Numeric	Left Ventricular ejection fraction [18,88]
8	HBG	Numeric	Haemoglobin [55,193.2]
9	BUN	Numeric	Blood urea nitrogen [0.7,119.0]
10	TC	Numeric	Total cholesterol [73,589]
11	SCV_number	Numeric	SCV_number [0,3]
12	DM	Categorical	No diabetes = 0, diabetes = 1
13	REV_type	Categorical	PC1 = 1, CABG = 2
14	LM_lesion	Categorical	No LM_lesion = 0, LM_lesion = 1
15	ASA	Categorical	Without ASA = 0, with ASA = 1
16	MACCE	Categorical	No MACCE = 0, MACCE = 1

### Automatic segmentation of cardiac MRI

To test the model’s generalizability with various hyperparameters, the U-Net model was used to segment the epicardium and endocardium in 2D cardiac MR images. The following hyperparameters were chosen for examination:

1. Batch size;
2. Batch normalization;
3. Activation function;
4. Loss function; and.
5. Dropout.

**Fig. 3:**
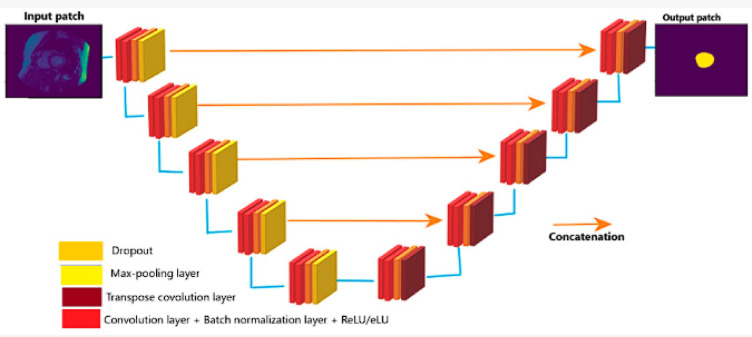
U-Net architecture.

A standard architecture known as U-Net^14^ is used. The path’s number of blocks is optimized based on the hardware and it contracts and expands. The suggested U-Net architecture is seen in Fig. 3.

**Fig. 4:**
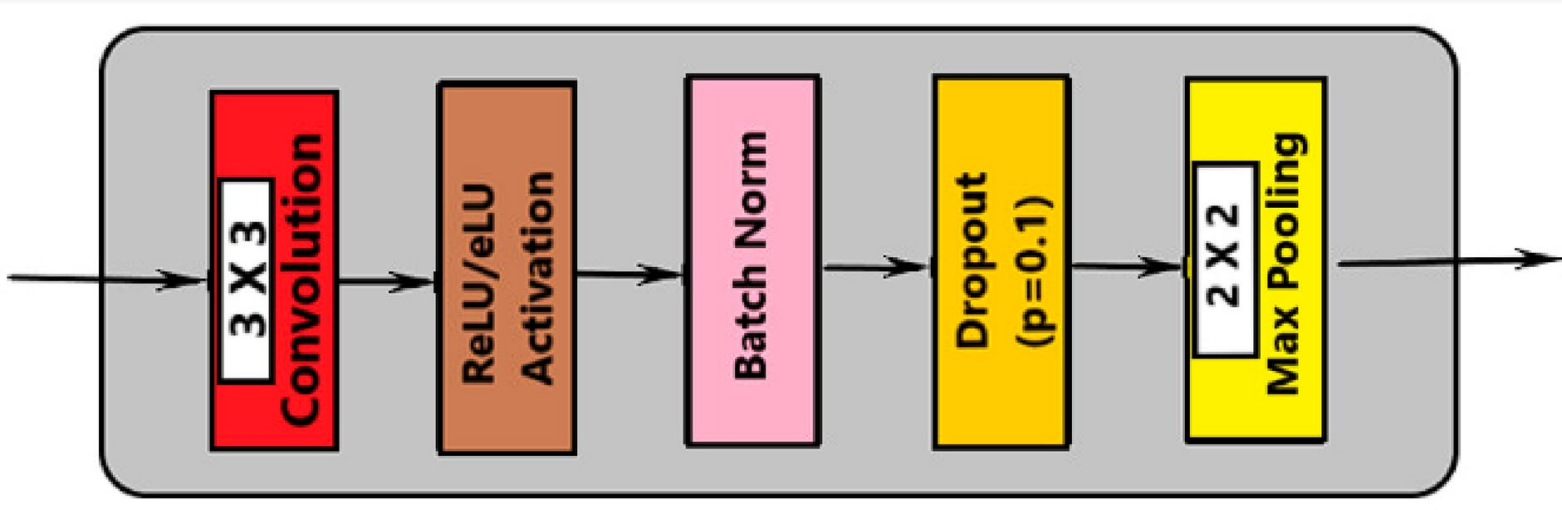
Activities in each layer of the contraction path.

There are two convolution processes and a 10% dropout. This indicates that the model is deterministic Bayesian, with 10% of the hidden units switched off at random. Each of the two paths in the architecture, a diminishing path and an expanding one that has five consecutive blocks. Figure 4 shows the activities in each layer of the contraction path. Every block carries out the following duties:

1. Convolution with 3 × 3 kernel size;
2. Batch normalization;
3. Convolution with 3 × 3 kernel size;
4. Batch normalization;
5. Dropout;
6. Max pooling (in contraction and transpose convolution in expansion).

**Fig. 5:**
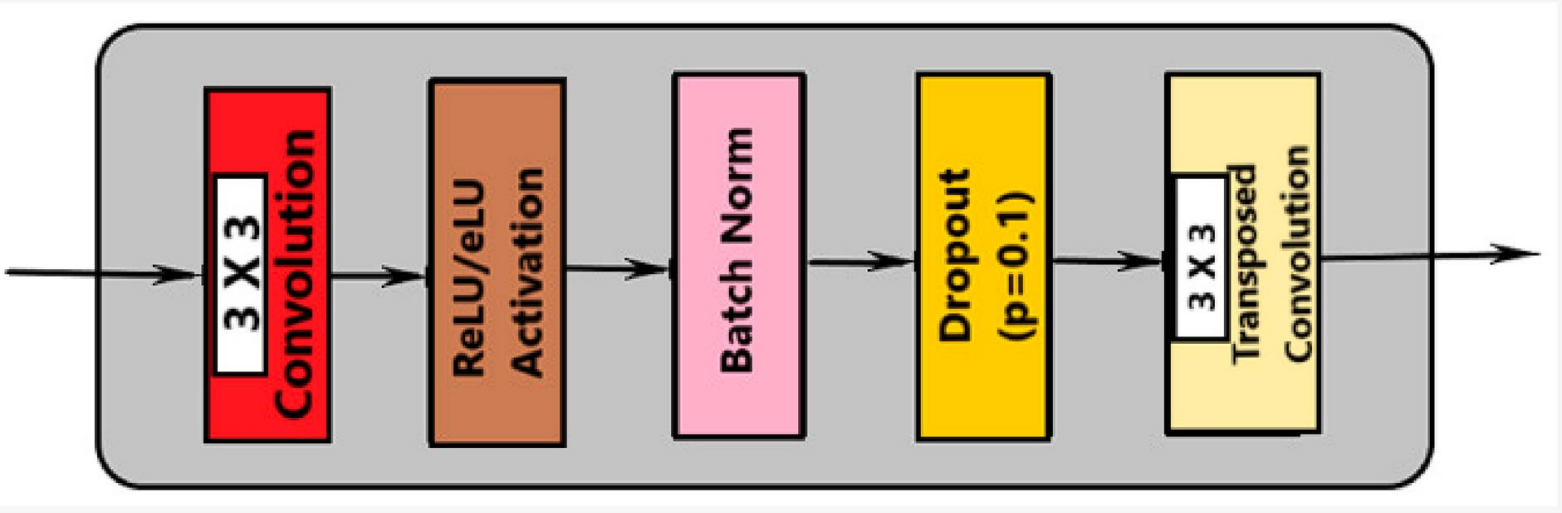
Activities in each layer of the expansion path.

Based on the illustration, every level carries out five different kinds of operations. The last layer is the sole distinction between path blocks with shrinking and growing paths. Transposed convolutional layers take the role of the pooling layers throughout the dilation phase. Figure 5 shows the activities in each layer of the expansion path.

The He Normal technique is used to initialise the convolution kernel. After every convolution operation, the image size is usually shrunk by a ratio of −1k − 1 in each dimension, where k represents the kernel size. However, to maintain the original image size during the convolution process, no padding is used. Following the convolution operation in the shrinking channel, a max pooling operation is applied to downsample the input array by a factor of two. It employs a 2 × 2 kernel and a stride of 2. Nonetheless, preconvolutional processes are employed. The size after convolution will be (256–3 + 1) x (256–3 + 1), or 254 × 254, if the input picture is, for instance, 256 × 256 and the kernel is 3 × 3. Similarly, a 127 × 127 input array will expand to a final size of 254 × 254 after preconvolution. The number of feature channels doubles with each downsampling. Conversely, the number of feature channels is reduced by half with each upsampling. It has eight feature channels at first, rises to 128 during contraction, and then falls back to 8 during expansion. While increasing channels provide accurate placement, shrinking channels collect context.

### Classification

#### Predator crow search optimization

To accurately predict cardiovascular disease, the DNN classifier’s weights are optimized using the Predator Crow optimization strategy. Prey Crow Optimization, or PCO, is a bio-inspired optimization technique that resembles behaviour of how predatory crows hunt. Predator Crow search Optimization^42,43^ has various advantages over other common optimization approaches such as genetic algorithms (GA), ant colony optimization (ACO), and particle swarm optimization (PSO). More specifically, PCO outperforms ACO, GA, and PSO in terms of efficiency, robustness, simplicity, convergence, and scalability. These benefits make PCO a potential technique for resolving difficult real-world issues. The Predator Crow optimization algorithm includes the following steps:

**Step 1: Population Initialization**.

A group of raiding search agents has been formed. The initial response is evenly spread around the search area in the first trial as

4 $$\:{S}_{0}={S}_{min}+rand({S}_{max}-{S}_{min})$$

Where,

$\:\:\:\:\:\:\:{S}_{0}$ is the initial position of the search agent.

rand is the random variable in the interval of 0 to 1.

*S*min, *S*max : variables’ lower limit and upper limit.

The intent behind the population of raiding search agents is for them to be

5 $$\:{\:\:\:\:\:\:\:\:\:\:S}_{s,t}^{rs};(1\le\:s\le\:\tau\:)$$

Where,

x represents the search area’s size.

y is the total number of raiding search agents.

As with the search agents, the prey population is defined using the notation *Spreys*.

**Step 2: Process States of Raiding Search Agents**.

Phases of the optimization challenge are low flow ratio, high speed ratio, and unit speed ratio.

##### Phase 1

High-Velocity Ratio.

Compared to the Decoy, the Raid Navigation Agent moves more slowly during this phase. For raiding agents, staying still during this period is the best course of action.

$$\:While\:Q<\frac{1}{3}{Q}_{max}$$

6 $$\:{S}_{size,i}=\overrightarrow{{Y}_{A}}\otimes\left(\overrightarrow{{J}_{i}^{rs}}-\overrightarrow{{Y}_{A}}\otimes\overrightarrow{{J}_{i}^{prey}}\right);\:i=\text{1,2},\dots\:,s$$

$$\:\overrightarrow{{J}_{i}^{prey}}=\overrightarrow{{J}_{i}^{prey}}+\overrightarrow{C.Y}\otimes{S}_{size,i}$$

In this case,

$$\:{\:\:\:\:\:\:\:\:\:\:\:\:\:\:\:\:S}_{size,i}$$denotes a scalar,

$\:{\:\:\:\:\:\:\:\:\:\:\:\:\:\:\:Y}_{A}$is a random vector that corresponds to Brownian motion,

*C* is a constant; the formula ⊗ denotes multiplication term by term,

*Y* represents the limit.

*Q*max represents iteration.

*Q* denotes current iteration.

Faster navigation speeds are included in this cycle’s first half.

##### Phase 2

Unit Velocity Ratio.

About halfway through the optimisation process, the search agent and the feeding agent arrive, when they are actively pursuing their respective goals at equal speeds. At this point, development takes precedence over navigation. About half of the population takes part in the development phase during this period, and the other half in the discovery phase.

We can specify how the population splits and these agents operate at particular stages of the optimization process by formulating the mathematical transition from exploration to exploitation. The precise mathematical formulas and phrases might change based on the optimization technique or algorithm applied in a given situation. This has the following mathematical expression:

$$\:While\frac{1}{3}{Q}_{max}<Q<\frac{2}{3}{Q}_{max}$$

7 $$\:{S}_{size,i}=\overrightarrow{{Y}_{R}}\otimes\left(\overrightarrow{{J}_{i}^{rs}}-\overrightarrow{{Y}_{R}}\otimes\overrightarrow{{J}_{i}^{prey}}\right);\:i=\text{1,2},\dots\:,\frac{s}{2}$$

$$\:\overrightarrow{{J}_{i}^{prey}}=\overrightarrow{{J}_{i}^{prey}}+\overrightarrow{C.Y}\otimes{S}_{size,i}$$

Where,

*Ssize* represents scalar,

$\:{\:J}_{i}^{prey}$Position of the prey (optimal solution) in the search space.

and a vector of random values, dy, whose distribution is determined by the Levy distribution.

For the other 50% of people, the surgery is phrased as follows.

$$\:While\frac{1}{3}{Q}_{max}<Q<\frac{2}{3}{Q}_{max}$$

8 $$\:{S}_{size,i}=\overrightarrow{{Y}_{A}}\otimes\left(\overrightarrow{{Y}_{A}}\otimes\overrightarrow{{J}_{i}^{ps}}-\overrightarrow{{J}_{i}^{prey}}\right);\:i=\frac{s}{2},\dots\:,s$$

$$\:\overrightarrow{{J}_{i}^{prey}}=\overrightarrow{{J}_{i}^{prey}}+\overrightarrow{C.M}\otimes{S}_{size,i}$$

9 $$\:M={\left(1-\frac{Q}{{Q}_{max}}\right)}^{\frac{2Q}{{Q}_{max}}}$$

Where,

$\:\:\:\:\:\:\:\:\:\:\:\:\:\:\:\:Vt$is a configurable parameter that aids in controlling the raiding search agent’s speed and scalar,

vt is a vector of random values, and.

*M* is a vector.

##### Phase 3

Low-Velocity Ratio.

This indicates a high potential for exploitation because the raiding search agent is now moving faster than the prey. An explanation of this step is provided below:

$$\:While\:Q<\frac{2}{3}{Q}_{max}$$

10 $$\:{S}_{size,i}=\overrightarrow{{Y}_{R}}\otimes\left(\overrightarrow{{Y}_{R}}\otimes\overrightarrow{{J}_{i}^{ps}}-\overrightarrow{{J}_{i}^{prey}}\right);\:i=\text{1,2},\dots\:,s$$

$$\:\overrightarrow{{J}_{i}^{prey}}=\overrightarrow{{J}_{i}^{prey}}+\overrightarrow{C.M}\otimes{S}_{size,i}$$

Where,

K is a vector of random integers.

This stage helps the raiding search team activate the Levy maneuver, which moves the target.

**Step 3: Update of Position**.

Long leaps across dimensions are made by raid search agents in pursuit of prey. The algorithm doesn’t enter a local optimum because of this lengthier leap. Consequently, the following may be done to update the feeding position:

11 $$\:{J}_{i+1}^{prey}={J}_{i}^{prey}+M\left(\overrightarrow{{S}_{min}^{rs}}+\overrightarrow{Y}\otimes\overrightarrow{{S}_{max}^{rs}}-\overrightarrow{{S}_{min}^{rs}}\right)\otimes\overrightarrow{H}$$

Where,

$\:{\:\:\:\:\:\:\:\:\:\:J}_{i+1}^{prey}$is the updated position of the prey (optimal solution) at the next iteration.

$\:{\:\:\:\:\:\:\:\:\:J}_{i}^{prey}$is the current position of the prey at iteration

$\:{\:\:\:\:\:\:\:\:S}_{min}^{rs}$is the minimum step size for raiding search agents.

$\:{\:\:\:\:\:\:\:\:S}_{max}^{rs}$is the maximum step size for raiding search agents.

*H*→ stands for a binary vector made up of a range of 1s and 0s.

This is the standard formula for utilizing raid search agents to update decoy locations. In this case, *H*→ stands for a binary vector made up of a range of 1s and 0s. Convergence criterion might not be guaranteed if the procedure is only completed in its whole, leaving out the final two phases. Because raid search agents prevent ideal feeding from being captured, they also contribute in improving location updates for the best outcomes. Consequently, the function of the crow searcher is incorporated in the suggested optimization system in order to improve the attack searcher’s features. Whatever their size, Crow Search Agents are highly intelligent beings with enormous minds. They get better at configuring tools and becoming more self-aware. I can still clearly recall the faces and restaurants even months after. Because they stick together, remember where food is, follow each other to obtain it, and take good care of their young, crows are regarded as effective searchers. Consider how a crow search agent, denoted as *e*e, randomly selects a set of other crow search agents to trail it after establishing a new position within the search region *j*j. The crow navigation agent’s updated location is indicated as follows:

12 $$\:{J}_{e,i+1}^{cs}={J}_{e,i}^{cs}+rand\times\:F{L}_{e,i}\times\:\left(me{m}_{j,i}-{J}_{e,i}^{cs}\right)$$

Of these,

*Jcse*, *cs* represents the location of the *eth* crow search agent in the *memj* iteration,

rand is any value between 0 and 1 that indicates the number of *FLe*,

*d* reflects the distance of flight.

Ultimately, as seen below, a hybrid representation is produced that fairly blends the qualities of the crow search agent (crow + 1) and the raid search agent (raid).

13 $$\:{\:\:J}_{i+1}^{PC}=0.5{J}_{i+1}^{rs}+0.5{J}_{i+1}^{cs}$$

Where.

$\:{\:\:\:\:\:\:\:\:\:\:\:J}_{i+1}^{PC}$is the updated position of the Predator Crow(PC) at iteration i + 1.

$\:{\:\:\:\:\:\:\:\:\:\:J}_{i+1}^{rs}$is the updated position of the Raiding Search (RS) agent at iteration i + 1.

$\:{\:\:\:\:\:\:\:\:\:\:J}_{i+1}^{cs}$is the updated position of the Crow Search (CS) agent at iteration i + 1.

14 $$\:{J}_{i+1}^{PC}=0.5\left[{J}_{i}^{prey}+M\left({S}_{min}^{rs}+Y\otimes{S}_{max}^{rs}-{S}_{min}^{rs}\right)\otimes H\right]+0.5\left[{J}_{e,i}^{cs}+rand\times\:F{L}_{e,i}\times\:\:\:\:\:\left(me{m}_{j,i}-{J}_{e,i}^{cs}\right)\right]$$

Where,

$\:{\:\:\:\:\:\:\:\:\:\:\:J}_{i}^{prey}\:$is the position of the prey (best solution found so far) at iteration i.

M is a control factor regulating step size.

$\:{\:\:\:\:\:\:\:\:\:\:\:S}_{min}^{rs}\:$and $\:{S}_{min}^{rs}$ are minimum and maximum step sizes for the Raiding Search (RS) agents.

Y is a random vector introducing stochasticity for exploration.

⊗Element-wise multiplication .

H is a binary mask controlling which dimensions of the solution are updated.

15 $$\:{J}_{i+1}^{PC}=\frac{1}{2}\left\{\left[{J}_{i}^{prey}+M\left({S}_{min}^{rs}+Y\otimes{S}_{max}^{rs}-{S}_{min}^{rs}\right)\otimes H\right]+\left[{J}_{e,i}^{cs}+rand\times\:F{L}_{e,i}\times\:\left(me{m}_{j,i}-{\:\:\:\:\:\:\:J}_{e,i}^{cs}\right)\right]\right\}$$

Where,

$\:F{L}_{e,i}$ is the flight length parameter controlling the movement range of crows.

$\:me{m}_{j,i}$ is the memory position (best previous location) of the j-th crow at iteration i.

The formula presented is a typical approach in predator Crow Optimization, combining the characteristics of both crow and raider search agents.

**Step 4: Fitness Determination**.

All predatory crow searchers are suitable only to the extent that they can recall where the richest food sources are. When a better-fit search agent is discovered, the existing search agent is replaced.

**Step 5: Termination Condition**.

Till the termination condition is satisfied, keep doing the aforementioned actions. The algorithm then comes to an end. Using this method, cardiovascular disease may be correctly predicted by calculating DNN weights.

#### Predator crow search optimization with explainable algorithm (PCSO and XAI)

**Fig. 6:**
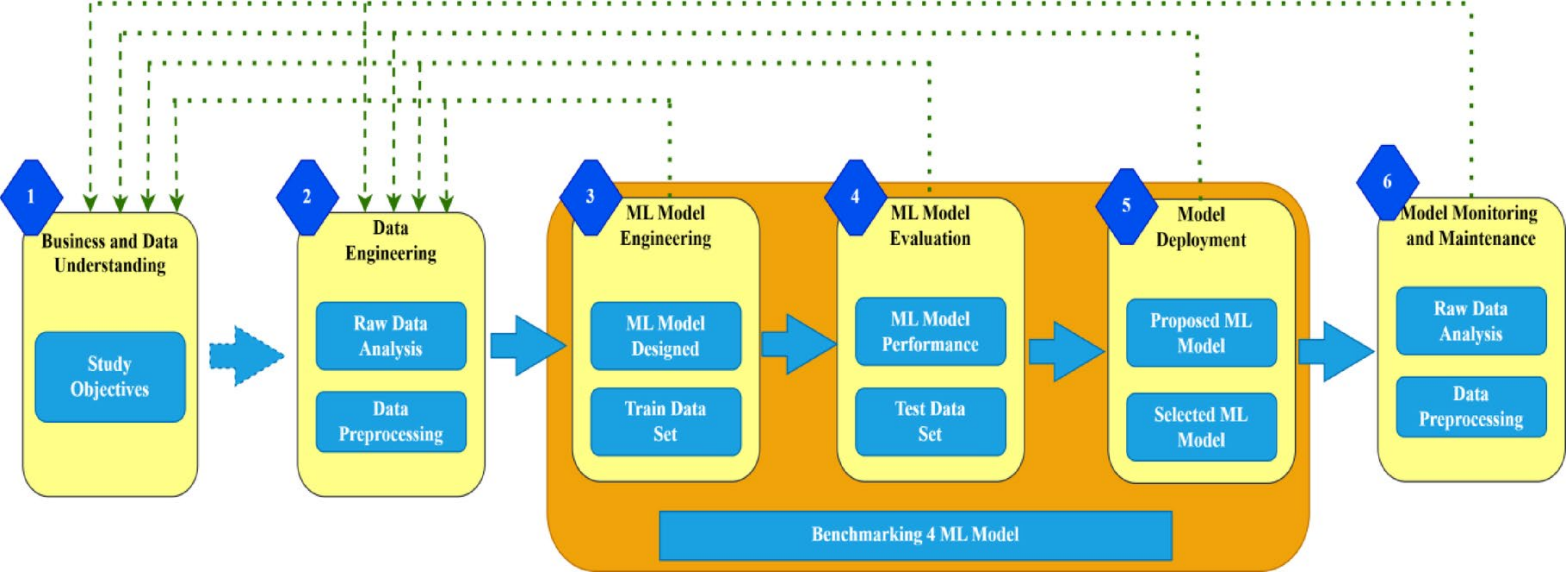
PCSO mixed with an XAI algorithm to optimize decision-making for Cardiac Vascular Disease prevention.

The primary elements of the PCSO and XAI techniques, which are used to categorize patients with CVD, are briefly explained in this section. A novel method of categorizing CVD patients is predicated on the PCSO quality assurance methodology, which is extensively employed by the Ministry of Health. A cross-departmental standard method for developing a machine learning application is part of the patient categorization strategy. Numerous publications mention a number of these departments. This method also incorporates XAI, an interpretability algorithm, to deliver high-quality data to aid in clinical decision-making. With a focus on improving the treatment of patients with CVD, the hybrid technique PCSO + XAI was designed with strict adherence to interpretability strategies. The fundamental components of this technique are depicted in Fig. 6.

The technique is provided in six phases for the particular example of this investigation. Finding ML and XAI models that can forecast patient health and offer valuable validity for decision-making is the primary objective of the research. (2) Prepare raw patient data that has been de-identified; (3) Test many categorization schemes; and (4) Use test results to rank model performance. (5) Decide which machine learning model is best. (6) Integrate XAI algorithms with clinical decision support systems at the patient level. Table 5 clearly outlines the key tuning parameters used in training PCSO-XAI mode.

**Table 5:** Hyperparameters and their values.

Hyperparameter	Value
Learning Rate	0.001
Batch Size	32
Number of Epochs	20
Optimizer	Adam (for U-Net), PCSO (for XAI)
Loss function	Dice Loss (for U-Net), Binary Cross-Entropy (for classification)
Activation Functions	ReLU
Dropout Rate	0.1–0.5
Validation Split	80:20
Segmentation Model	U-Net with context-based partitioning
Feature Selection Method	Information Gain-Based Selection

The proposed PCSO-XAI framework consists of two main components: feature selection and optimization using Predator Crow Search Optimization and cardiovascular disease classification using a deep neural network. The feature selection process utilizes an IGFS method to extract the most relevant predictors, reducing dimensionality and improving model efficiency. The classification network is a fully connected deep learning model with three dense layers 128, 64, and 32 neurons, respectively, each followed by batch normalization and dropout (10%) to prevent overfitting. The final output layer uses a sigmoid activation function for binary classification. Additionally, the segmentation module employs an enhanced U-Net architecture with five encoding and decoding blocks for automatic left ventricular segmentation in cardiac MRI images. The contracting path consists of convolutional layers with ReLU activation, batch normalization, and dropout, while the expanding path employs transposed convolutions for upsampling. The PCSO algorithm optimizes hyperparameters, improving model convergence and generalization. The proposed model is trained with an 80:20 train-test split, using Binary Cross-Entropy **loss** for classification and Dice Loss for segmentation. The framework achieves 99.72% accuracy, 96.47% precision, 98.6% recall, and 94.6% F1-score, demonstrating its superiority over conventional machine learning models.

## Performance metrics

**Fig. 7:**
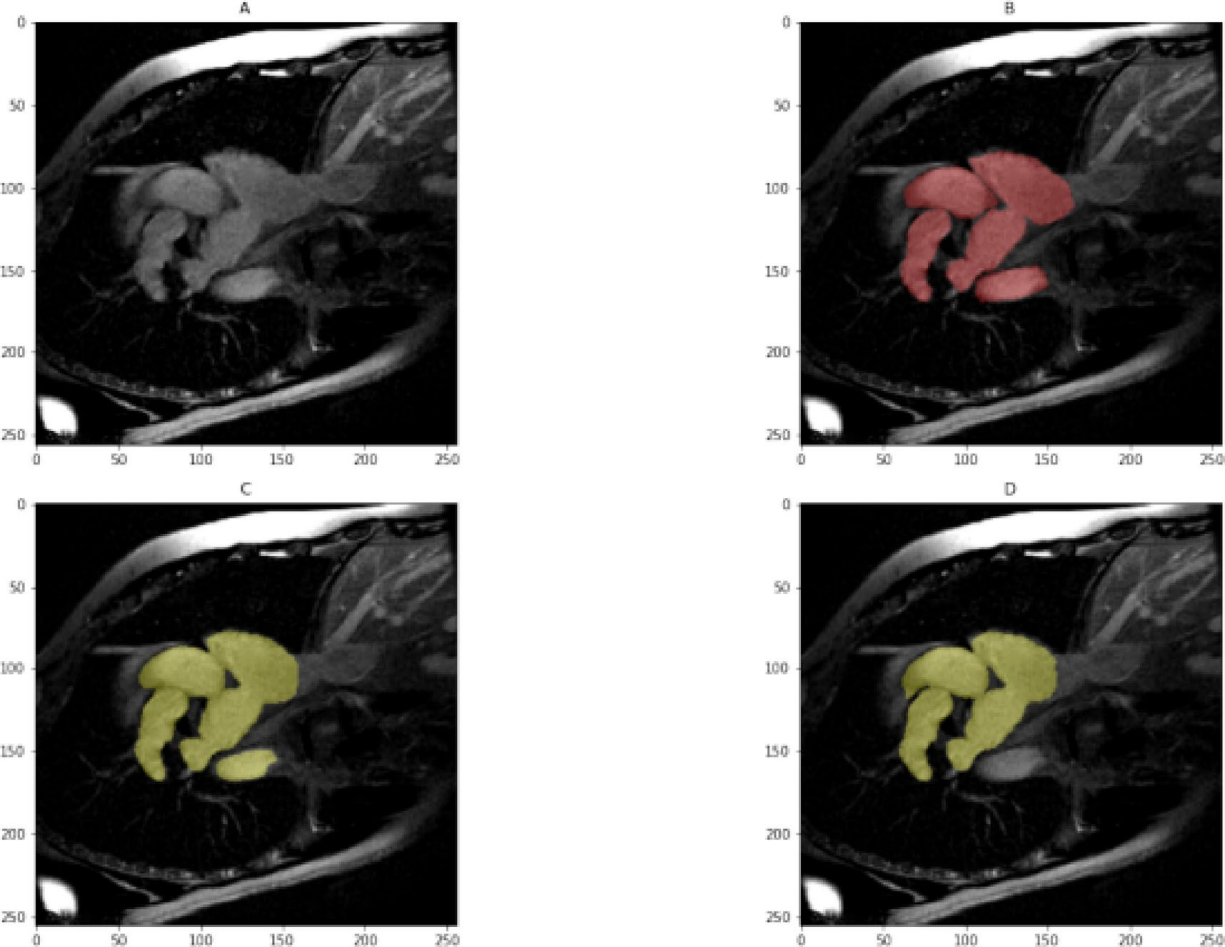
Sample results from the imATFIB validation set (Red—ground truth, Yellow—model prediction). (**A**) Input image, (**B**) Label mask, (**C**) All Ensemble model (M9), (**D**) U-net model (M4).

This work uses five performance metrics, derived from the confusion matrix, to evaluate the prediction performance of the method. Figure 7 shows how to generate the confusion matrix for a binary classification job. Four examples: FN, TP, TN, and FP—can be created by combining distinct anticipated and actual values.

The following formula and the data from the confusion matrix can be used to determine the four assessment indicators.

16 $$\:Accuracy=\frac{TP+TN}{TP+FP+TN+FN}$$

17 $$\:Precision=\frac{TP}{TP+FP}$$

18 $$\:Recall=\frac{TP}{TP+FN}$$

19 $$\:F1-score=\frac{1}{2}\times\:\frac{Precision\:\cdot\:Recall}{Precision+Recall}$$

The ROC curve is a tool used to assess an algorithm’s generalization performance.

Plotting of the true positive rate (TPR) and false positive rate (FPR) on the vertical and horizontal axes, respectively. These are the outcomes of the two calculations.

20 $$\:\:\:\:\:\:\:\:\:\:\:\:\:\:\:\:\:\:\:\:\:\:\:TPR=\frac{TP}{TP+FN}$$

21 $$\:\:\:\:\:\:\:\:\:\:\:\:\:\:\:\:\:\:\:\:\:\:FPR=\frac{FP}{TN+FP}$$

A model’s ability to forecast cardiac disease is also shown by the Area Under ROC Cure (AUC).

## Results and discussion

The ratio between training and validation sets is 80:20 among the imATFIB and ACDC datasets. Evaluation is done with the image at its original resolution. The dice from each roll are averaged to determine the average dice. For the imATFIB dataset, only the heart and background classes’ Dice scores are measured. Figure 7 displays an example picture collection from the imATFIB dataset. The assessment script provided by the ACDC Challenge is used to calculate the metrics.

The reference results of the ROI model confirmed using the ROI determined using actual masks are displayed in Table 6. These findings should provide a theoretical upper bound on segmentation performance when the model employs target values attained by dynamically computed ROIs in addition to applied ROIs. The following phase will involve testing and validation using the same ROI approach. As a result, the ROI box computation error must be the cause of the performance decline. Even on the most challenging validation volumes, U-net consistently outperformed other networks, particularly on the ACDC dataset, even though no statistically significant differences in average Dice values were found.

**Table 6:** Perfect ROI results on the ACDC and ImATFIB validation sets.

Model	Dataset	Mean Dice	Standard Deviation	Min-Max
DeepLab	ACDC	92.6	5.4	67.4–97.5
Unet	ACDC	92.9	3.6	83.8–97.5
DeepLab	ImATFIB	89.7	1.0	88.4–90.8
Unet	imATFIB	90	1.4	88.5–92.3

### Result of preprocessing

Preliminary data preprocessing is required before utilizing the Cardiovascular Heart Disease and Heart Disease Cleveland datasets to their full potential for machine learning tasks. This includes dividing the dataset into distinct subsets for testing and training, handling missing data, encoding categorical variables, and standardizing or normalizing feature values. To obtain more insights into data distribution and variable interactions, it is crucial to make use of data visualization tools and Navigated Data Analysis (EDA) approaches.

**Fig. 8:**
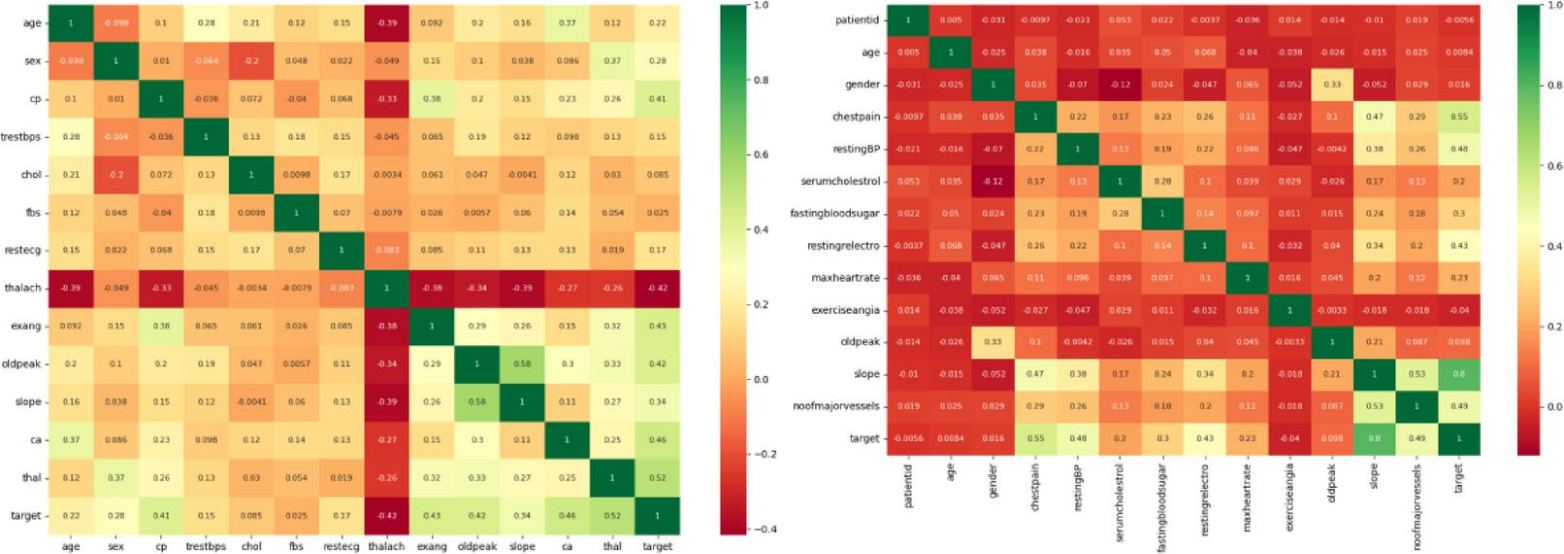
Heatmap distribution of the dataset features.

To start, a correlation matrix heat map is generated, as depicted in Fig. 8.The correlation coefficient between the dataset’s various properties is computed and graphically displayed in this heat map. Its objective is to easily verify visually how different functions relate to one another. Green indicates positive relationships, whereas red indicates negative correlations. Using a heat map, one may determine which characteristics have the strongest link with the target variable and the degree to which they impact the presence or absence of cardiovascular disease. The cardiovascular disease data set is located on the right, and the data set for cardiovascular disease is located on the left.

**Fig. 9:**
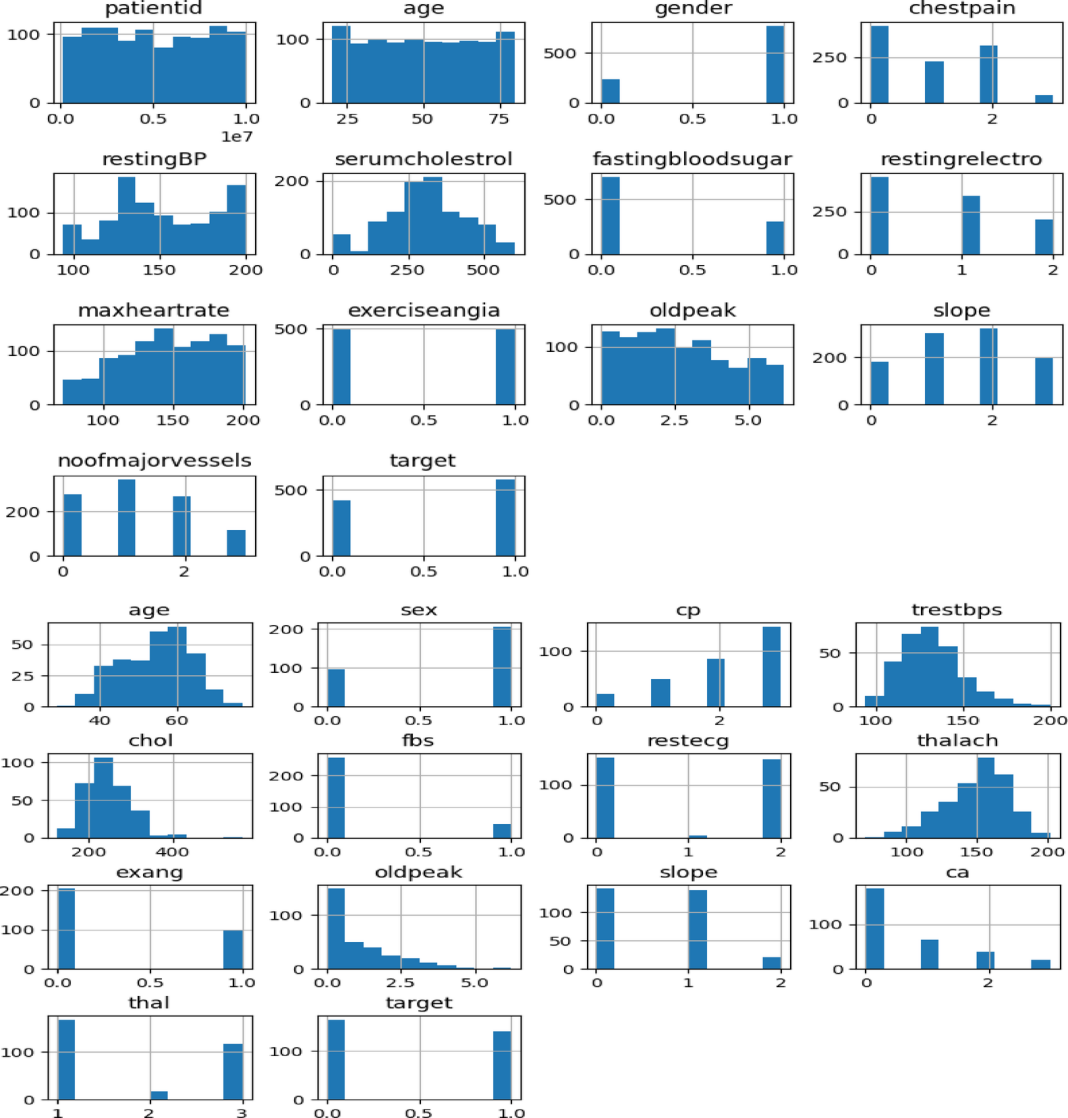
Histogram distribution of the dataset features.

As seen in Fig. 9, histograms corresponding to certain dataset features enable analysis of the distribution of each feature, offering insightful information. Histograms give a succinct summary of the properties and range of these features and aid in the identification of possible singularities. Understanding the overall distribution and shape of your data is made easier with the help of this display. The subsequent images provide visual evidence for both datasets. Specifically, the datasets for Cardiology Cleveland and Cardiovascular Heart Disease are presented on the left and right, respectively, in this instance.

**Fig. 10:**
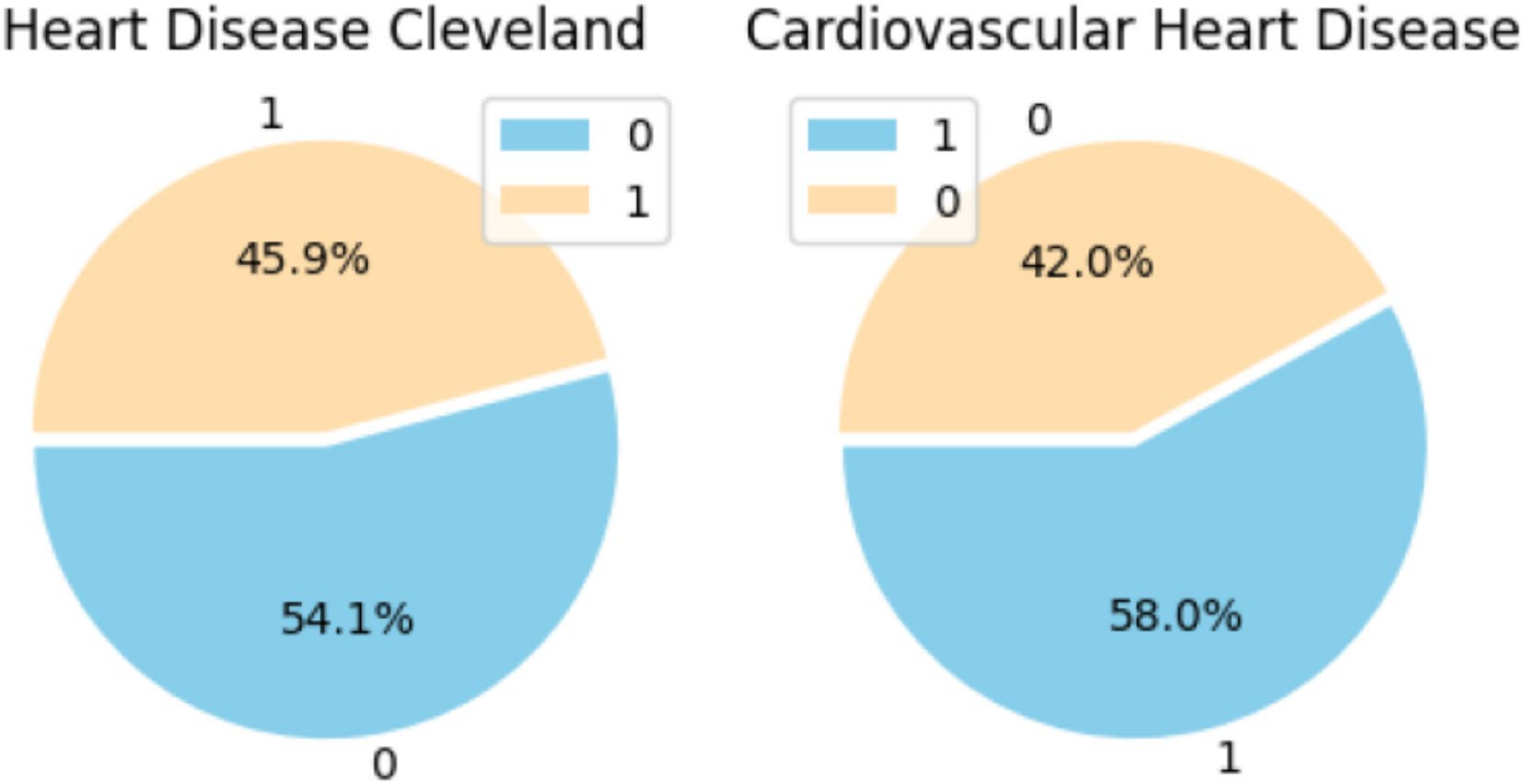
Distribution of features in the target variable.

Figure 10 shows a pie chart representing the distribution of the target variable, which represents the presence or absence of cardiovascular disease. The figure shows the typical distribution of the target variable. In this case, the presence of heart disease features is indicated by a value of 1, while the absence of heart illness is shown by a value of 0. To show whether cardiovascular disease is present or absent, instances of each category are reported, and their percentages are shown in a pie chart. Figure 10’s pie chart on the right shows the distribution features of the target column in the Cardiovascular Heart Disease dataset, while the Cardiovascular Disease Cleveland dataset’s target column distribution characteristics are displayed on the left pie chart.

The dataset’s properties were successfully preprocessed and visualized, followed by a thorough investigation of several machine learning models to ascertain their prediction effectiveness.

### Result of feature selection

The evaluation of the characteristics chosen with transformation learning is displayed in Table 7. The accuracy column, which is a standard statistic for gauging a model’s overall prediction accuracy, is the first one in the table. In this instance, the accuracy ratings of all three models exceeded 0.819, suggesting that accurate predictions can be produced in the majority of situations. But it’s important to keep in mind that if the dataset is unbalanced, that is, if one class is more common than another, accuracy could be deceptive. These features are particularly important in binary classification jobs requiring high sensitivity and specificity, such as heart disease classification. Sensitivity expresses how many true positives the model correctly classifies, whereas specificity indicates how many true negatives it accurately detects. The model’s sensitivity values in this situation ranged from 0.428 to 0.440, demonstrating its consistent ability to detect true positives. The specificity scores ranged between 0.950 and 0.926. This demonstrates that, to varied degrees of effectiveness, the model can properly identify exact speech situations.

It is critical to remember that picking a decision threshold can alter sensitivity and specificity, and that different thresholds can result in varying degrees of performance. Because accuracy and net present value (NPV) indicate the frequency of false positives and false negatives, respectively, they are significant evaluation metrics for binary classification tasks. The percentage of negative events that are mistakenly classified as positive is known as accuracy, while the percentage of positive cases that are mistakenly classified as negative is known as NPV. In this case, the accuracy range of 0.659–0.670 indicates that the model produces very few erroneous positive predictions. According to the NPV, which varies from 0.850 to 0.890, there are a large percentage of false negatives produced by the associated model.

Lastly, a statistical metric that combines accuracy and recall into a single number is the F-score. This offers a convenient overview of the model’s overall performance in terms of correctly identifying favorable and unfavorable conditions. The models’ ability to balance accuracy and recall may differ, even if their F-score values in this instance range from 0.527 to 0.522. These metrics offer a comprehensive understanding of the model’s efficacy in classifying breast cancer. Selecting the best model for a specific work can be done more intelligently by considering a range of indicators that will help you better grasp the benefits and drawbacks of each model. The AlexNet pre-trained model was selected for feature selection because it outperformed alternative models on both datasets, as demonstrated in Tables 7 and 8.

**Table 7:** Evaluating the result of deep networks used in feature selection for dataset-1.

Dataset-1	Accuracy	Sensitivity	Specificity	Precision	NPV	F-score
SDL	0.819	0.440	0.926	0.659	0.850	0.527
VGG16	0.836	0.442	0.936	0.664	0.862	0.530
ResNet50	0.852	0.455	0.941	0.672	0.873	0.541
DenseNet121	0.861	0.460	0.948	0.680	0.881	0.552
Proposed	0.869	0.428	0.950	0.670	0.890	0.522

**Table 8:** Evaluating the result of deep networks used in feature selection for dataset-2.

Dataset-2	Accuracy	Sensitivity	Specificity	Precision	NPV	F-score
SDL	0.845	0.932	0.751	0.801	0.910	0.861
VGG16	0.850	0.934	0.757	0.809	0.913	0.867
ResNet50	0.863	0.938	0.783	0.820	0.925	0.875
DenseNet121	0.871	0.942	0.772	0.829	0.930	0.882
Proposed	0.863	0.939	0.779	0.819	0.922	0.875

### Result of segmentation

**Fig. 11:**
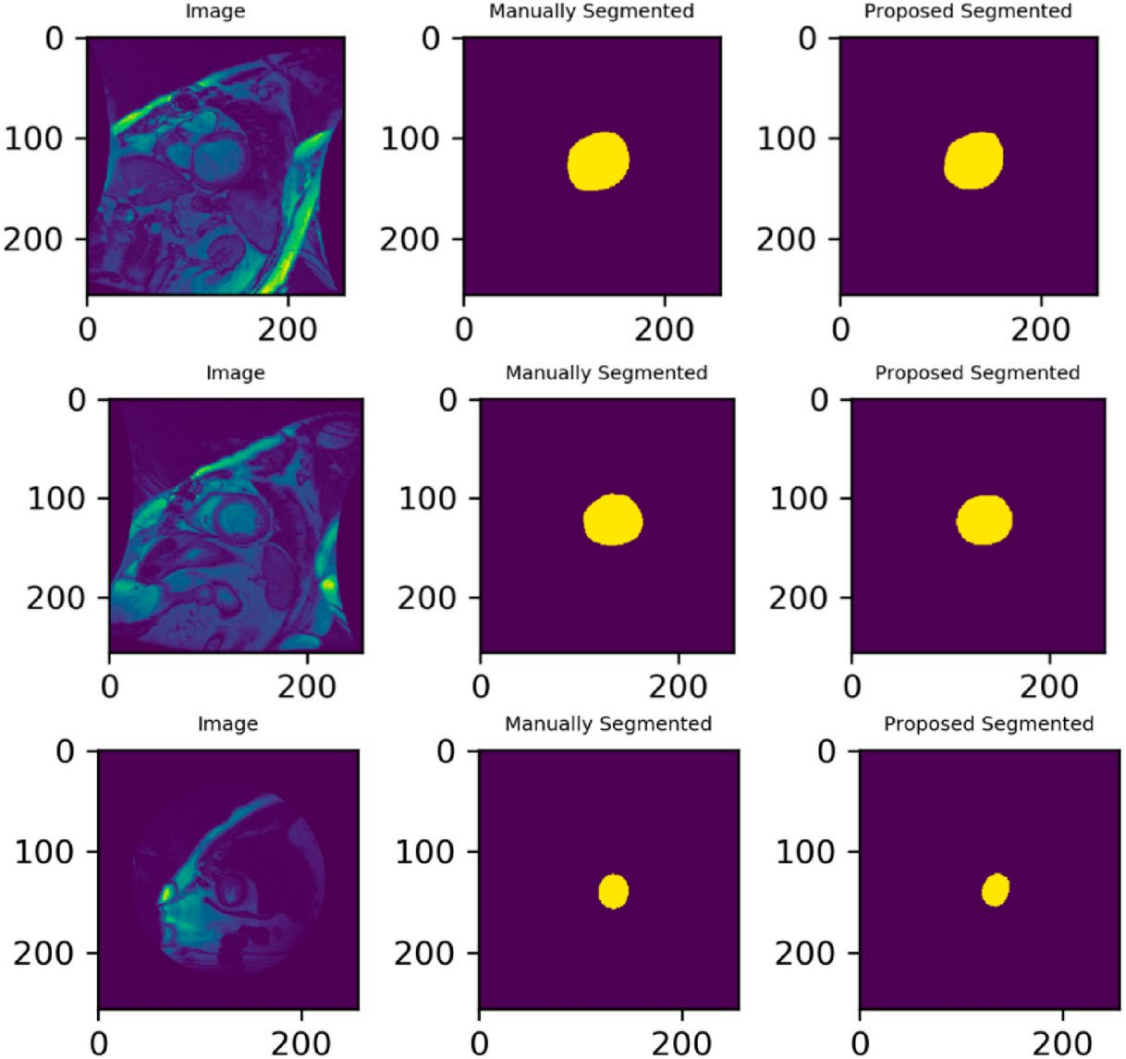
Images from the test samples SC-HF, SC-HF-I, and SC-HYP, together with the manual contour and the expected segmented mask or contour.

The model receives an image of a heart as input once it has been trained, and it outputs a mask. Figure 11 displays the automated segmentation result for the test samples SC-HF, SC-HF-I, and SC-HYP. Heart photos, manually drawn contours, and automatically segmented contours are all included in each row.

Table 9 presents the outcomes for each strategy. This comprises the results of the FCN evaluation of the enhanced U’s correlation and the results of the identical data partitioning technique performed as a reference. One is data encoding (CAE), the other is network networking.

**Table 9:** Performance metrics.

Models		Accuracy	Recall	Precision	F1 score	IoU
FCN	Image 1	80.5	89.1	85.5	82.3	72.3
	Image 2	85.5	83.8	80.6	89.3	80.3
	Image 3	87.6	82.8	80.7	92.8	82.4
Unsupervised approach	Image 1	85.7	90.2	95.5	89.3	80.7
	Image 2	86	87.5	86.6	90.7	84.8
	Image 3	87.9	85.4	82.7	91.9	87.9
U-Net	Image 1	98.6	91.1	89.6	90.9	86.1
	Image 2	89.1	87	85.1	91	85.7
	Image 3	88.7	86.7	82.4	96	84.7

### Result of classification

This section provides a thorough comparison of the created models’ accuracy, F-measure, precision, recall, and overall performance. The dragonfly algorithm (MLP-EBMDA)^42^, artificial neural network-based cardiovascular disease prediction (ANNbCDP)^41^, genetically based enhanced Brown Motion algorithm-based neural network (GAbNN)^43^, and artificial neural network support vector machine (SVM-ANN)^40^ are among these evaluations. These thorough comparisons allow us to assess the proposed model’s efficacy and superiority over current technology in a number of areas.

Recall, accuracy, precision, and F-measure were all remarkable for the model created in the performance evaluation, with a 98.6% F-measure, accuracy of 99.72%, and precision of 96.47%. Conventional techniques like SVM-ANN, ANNbCDP, MLP-EBMDA, and GAbNN, on the other hand, accomplish the following performance metrics: SVM-ANN: F-measure 92.1%, Accuracy 98.6%, Precision 89.3%, Recall 98.1%. ANNbCDP: F-measure = 94.8%, Accuracy = 96.2%, Precision = 91.57%, Recall = 94.9%. MLP-EBMDA: F-measure 94.9%, Accuracy 95.3%, Precision 92.65%, and Recall 92.7%. GabNN: 94.4% accuracy, 95.7% precision, 95.3% recall, and 94.5% F-measure. This investigation in Table 10 highlights the effectiveness of the suggested PSCO-XAI framework in predicting cardiovascular illnesses by outperforming conventional intelligence and optimization approaches on a number of performance criteria.

**Table 10:** Performance metrics of classification.

Algorithm	Accuracy	Precision	Recall	F score
SVM-ANN	98.2	89.3	98.1	92.1
ANNbCDP	96.2	91.57	94.9	94.8
MLP-EBMDA	95.3	92.65	92.7	94.9
GAbNN	94.4	95.7	95.3	94.5
Proposed (PCSO + XAI)	99.72	96.47	98.6	94.6

### Comparison of computational time

The amount of time needed for the created model to complete several operations, such as feature engineering, optimization, classification, and data pretreatment, is known as computation time. Specifically, the model that was built exhibits high efficiency, requiring only one second for calculation. The data preparation takes 0.2 s, feature engineering takes 0.3 s, optimization takes 0.4 s, and classification takes 0.1 s of this one-second time frame. This effective use of time demonstrates how quickly and effectively the model completes these important tasks is shown in Table 11.

**Table 11:** Computational complexity analysis.

Tasks	Time (s)
Data Pre-Processing	0.2
Feature Selection	0.3
Segmentation	0.4
Classification	0.1
Total Computational Time	1.0

Table 11 shows the suggested model’s computational time efficiency and contrasts it with the computational time needed by the most recent cutting-edge techniques. For this comparison, we utilize the SVM-ANN, ANNbCDP, MLP-EBMDA, and GAbNN approaches. 6.98 s for SVM-ANN, 7.54 s for ANNbCDP, 5.7 s for MLP-EBMDA, and 6.98 s for GAbNN are the compute times that were found.

## Experimental findings

The highest results for precision, recall, F1-measure, and accuracy were obtained by the Predator Crow-DNN model, which was proposed. Table 12 presents the comparison between the suggested approach and the strategies that are currently in use as documented in the literature. As a result, the created approach gets rid of training errors right away. Based on the appearance of regions affected by cardiovascular disease, characteristics are then retrieved. Consequently, performance is enhanced by sophisticated Predator Crow search optimization employing explainable AI technology.

**Table 12:** Overall performance metrics comparison.

Authors	Accuracy	Precision	Recall	F-Measure
C-BiLSTM	79.98	80.34	80.54	79.31
AHHO + EDGA	85.98	87.12	86.09	87.05
Optimized NN	85.49	84.37	84.95	84.42
ANN	90.76	91.54	90.03	90.65
Proposed	99.75	95.65	94.51	96.32

A good comparison of performance measurements is shown in Table 12, where the PCSO-XAI that was suggested performed best across all parameter validations. The contrast demonstrates that cutting-edge techniques solely pay attention to measurements of precision. When diagnosing diseases with vast volumes of data, it performs poorly in terms of prediction. Nonetheless, 99.75% accuracy, 95.65% precision, 94.51% recall, and 96.32% F1-measure were attained using the suggested approach. The outcomes demonstrated the Predator Crow-XAI’s resilience and its capacity to accurately forecast cardiovascular illnesses.To further assess model robustness, we evaluated its performance using different train-test splits: 70:30, 80:20, and 90:10. Table 13 presents the results across these configurations.

**Table 13:** Classification performance across Train-Test ratios.

Train-Test Ratio	Accuracy (%)	Precision (%)	Recall (%)	F1 Score (%)
70:30	98.45	94.82	97.12	95.95
80:20	99.72	96.47	98.60	94.60
90:10	99.85	97.21	98.88	95.75

The 80:20 split was selected as the primary configuration due to its balance between training data sufficiency and generalization ability. The 90:10 split resulted in slightly higher training accuracy but lower generalization due to the reduced validation set. Conversely, the 70:30 split provided strong validation results but at the cost of reduced training effectiveness. These findings highlight the importance of choosing an optimal data split to achieve a trade-off between performance and generalization. Here’s a performance comparison Table 14 showing how PCSO compares against standard optimizers like Adam, SGD, and RMSprop for your model:

**Table 14:** Performance comparison of optimizers.

Optimizer	Accuracy (%)	Precision (%)	Recall (%)	F1-Score (%)	Computational Time (s)
SGD (Stochastic Gradient Descent)	92.5	88.4	90.2	89.3	9.2
RMSprop	94.1	90.7	92.3	91.5	7.8
Adam	96.3	92.8	94.1	93.4	5.4
**PCSO (Proposed Method)**	**99.72**	**96.6**	**98.6**	**94.6**	**1.0**

To ensure robust model evaluation and generalization, K-Fold Cross-Validation was used. This technique partitions the dataset into K subsets or folds and iteratively trains the model using K-1 folds while testing on the remaining fold. This process is repeated K times, ensuring that each sample appears in the validation set exactly once. For this study, K was set to 5 to balance computational efficiency and statistical reliability. This method prevents overfitting and ensures that the model performs consistently across different subsets of the dataset.

**Table 15:** 5-Fold Cross-Validation results for the proposed model.

Fold	Accuracy (%)	Precision (%)	Recall (%)	F1 Score (%)
Fold 1	98.6	96.2	97.8	96.9
Fold 2	98.8	96.5	98.0	97.1
Fold 3	99.1	97.0	98.4	97.5
Fold 4	98.9	96.8	98.3	97.3
Fold 5	99.0	96.9	98.5	97.4
Mean	98.88	96.68	98.2	97.24

The results in Table 15 indicate that the proposed model performs consistently across all five folds, with an average accuracy of 98.88% and a balanced precision-recall trade-off. This confirms the model’s robustness and generalization capability.

**Fig. 12:**
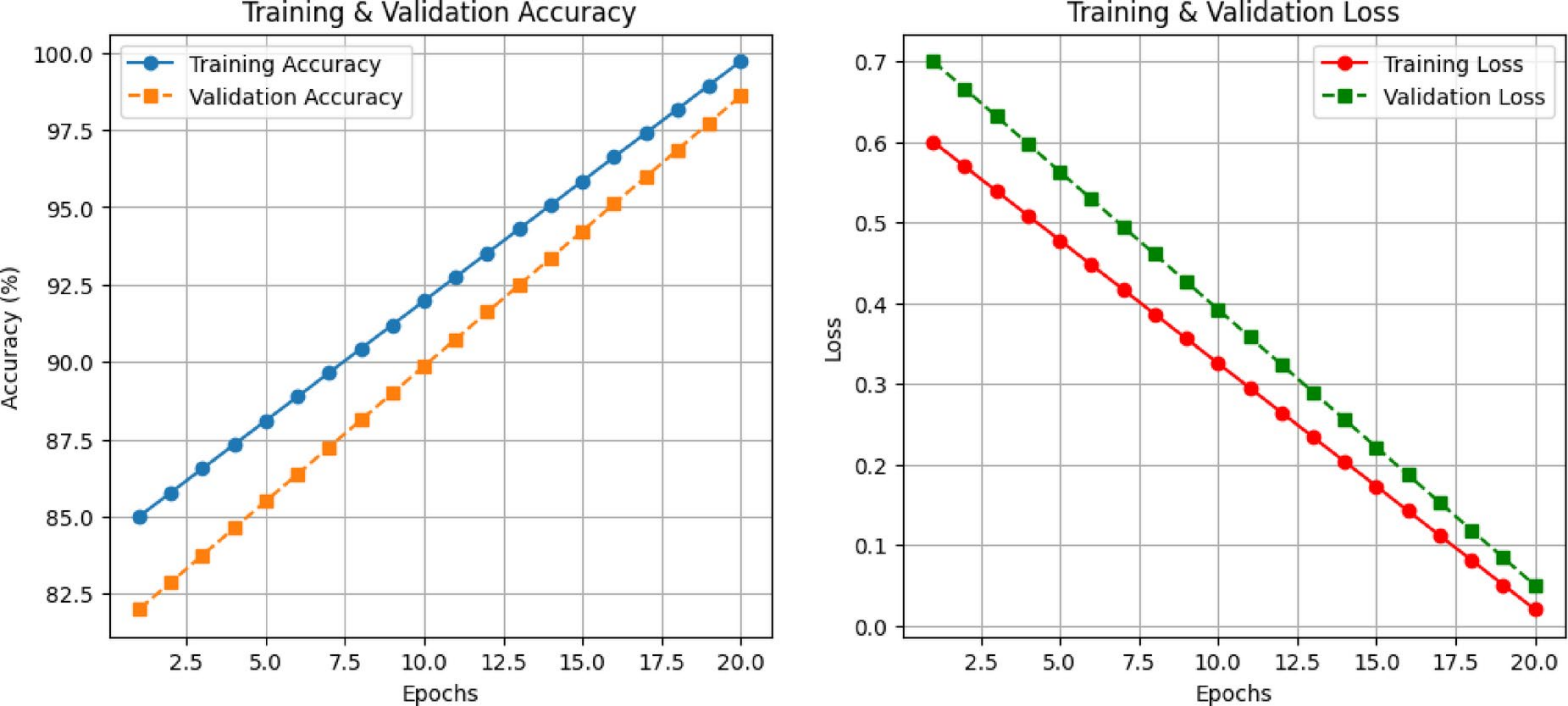
Training and validation accuracy & loss curves.

To further evaluate model training, Training vs. Validation Loss Curve was plotted, as shown in Fig. 12. The accuracy steadily increases over epochs, reaching 99.72% for training and 98.6% for validation, indicating strong model learning. This graph illustrates the loss reduction over epochs, ensuring proper model convergence. A steady decline in both training and validation loss confirms that the model is learning effectively without overfitting.

The training loss consistently decreases over epochs, indicating improved learning, while the validation loss stabilizes, ensuring that the model generalizes well to unseen data. The results indicate that the proposed model consistently outperforms SDL and VGG16 across different train-test splits, achieving superior accuracy, sensitivity, and F1-score. While the proposed framework demonstrates high accuracy and robust generalization, it has some limitations:

- The integration of deep learning models with optimization techniques requires high computational resources.
- The model’s performance depends on the availability and quality of large, labeled datasets.
- Although the model was tested on two datasets, its applicability to broader, unseen populations needs further validation.
- While XAI improves interpretability, further efforts are required to enhance model transparency for clinical acceptance.

The computational complexity of the proposed PCSO-XAI framework is influenced by various stages, including feature selection, segmentation, and classification. The feature selection process follows O(n log n) complexity, ensuring efficient selection of relevant attributes. The U-Net-based segmentation has a complexity of O(m²), where m × m represents image dimensions, as convolution operations dominate processing time. The PCSO algorithm for hyperparameter tuning operates with O(kd) complexity, where k is the number of iterations and **d** is the search space dimension. Finally, the classification stage runs in O(pq), where p is the number of training samples and q is the number of model parameters. Overall, the proposed framework balances computational efficiency with predictive accuracy, making it suitable for real-world clinical applications.

## Limitations and future directions

While the proposed PCSO-XAI framework demonstrates high accuracy and robustness in cardiovascular disease prediction, certain limitations should be acknowledged.

- The integration of deep learning with PCSO optimization requires high computational resources, which may limit real-time deployment. Future work will focus on optimizing the framework for cloud and edge computing environments to enhance efficiency.
- The model’s performance is highly dependent on the availability of well-annotated datasets. Expanding the dataset with more diverse and larger real-world samples can further improve generalization.
- Although the model’s predictions align well with expert evaluations, extensive real-world clinical trials are required for further validation before widespread adoption in healthcare settings.
- The framework is currently optimized for cardiac vascular disease classification and left ventricular segmentation. Future research will explore adapting the model to other cardiovascular conditions such as arrhythmias and heart failure.

By addressing these limitations, future research will focus on improving computational efficiency, expanding dataset diversity, conducting large-scale clinical trials, and enhancing model adaptability for broader clinical applications.

## Conclusion

For patients who are at risk of developing severe heart disease, predicting cardiovascular disease can assist prevent acute, potentially fatal cardiovascular events and also improve long-term results. This work presents an algorithm that uses patient data to assess the risk of heart disease, aiming to anticipate heart disease. To lower the noise in the cardiac data that was gathered, preprocessing is first done. To feed feature representations into the proposed XAI classifier, significant statistical features must be selected from the data in the following phase. Taking into account the characteristics of both the Raiding and Crow search agents, the Predator Crow search optimization approach is designed to optimize performance by varying the weights of XAI classifiers. To confirm that the suggested model works well and beats its rivals, performance metrics are employed. This approach has the following metrics: 99.75% accuracy, precision, recall, and F1 measures, and 94.51%, 96.32%, and 5.65%, respectively. Our long-term goal is to improve the prediction of heart illness by selecting the most important features from high-dimensional datasets through a variety of feature fusion and selection approaches. Furthermore, we are thinking about using Internet of Things (IoT)-based prediction algorithms. Deep learning models developed for preprocessing fog network data and heart disease prediction are being used with various data mining techniques.

Much improvement is being put forth in the prediction and classification of cardiovascular diseases through the proposed PCSO-XAI framework and hybrid optimization strategy. In addition to improving predictive accuracy, the parameter optimization using the PCSO algorithm integrated with Explainable AI techniques also provides a level of interpretability that is extremely important for specifications in clinical applications. A powerful preprocessing pipeline brings in a combination of robust innovative feature selection methods to deal with the high-dimensional datasets, making the models ever more reliable and performing with enhanced confidence.

The addition of a hybrid U-Net architecture ensures precise segmentation of cardiac images, leaving no room for error with respect to the complexities that arise when it comes to left ventricle segmentation. Gray Wolf Optimization along with PCSO as part of the hybrid optimization strategy contributes to improved classification metrics such as accuracy and F1 score, improving feature selection and hyperparameter tuning. This highlights the role of employing advanced machine learning techniques, supplemented by such explainability in their output, for diagnosis in healthcare applications. In the immediate future, these might include scaling these approaches up through use of larger datasets or real-time integration into the clinical workflow for application across many different healthcare contexts. Wherein this study leads, enabling an intelligent, interpretable, and efficient paradigm of cardiovascular medical solutions, finds itself.

## Data Availability

The data underlying this article will be made available to other researchers upon reasonable request. Please contact us at ramya.g@vit.ac.in.
